# A geometrical model for testing bilateral symmetry of bamboo leaf with a simplified Gielis equation

**DOI:** 10.1002/ece3.2407

**Published:** 2016-09-01

**Authors:** Shuyan Lin, Li Zhang, Gadi V. P. Reddy, Cang Hui, Johan Gielis, Yulong Ding, Peijian Shi

**Affiliations:** ^1^ Collaborative Innovation Center of Sustainable Forestry in Southern China of Jiangsu Province Bamboo Research Institute College of Biology and the Environment Nanjing Forestry University Xuanwu District Nanjing China; ^2^ Western Triangle Ag Research Center Montana State University Conrad MT USA; ^3^ Centre for Invasion Biology Department of Mathematical Sciences Stellenbosch University Matieland South Africa; ^4^ Mathematical and Physical Biosciences African Institute for Mathematical Sciences Cape Town South Africa; ^5^ Department of Biosciences Engineering University of Antwerp Antwerp Belgium

**Keywords:** bamboo, bilateral symmetry, goodness of fit, leaf‐shape parameter, polar coordinate

## Abstract

The size and shape of plant leaves change with growth, and an accurate description of leaf shape is crucial for describing plant morphogenesis and development. Bilateral symmetry, which has been widely observed but poorly examined, occurs in both dicot and monocot leaves, including all nominated bamboo species (approximately 1,300 species), of which at least 500 are found in China. Although there are apparent differences in leaf size among bamboo species due to genetic and environmental profiles, bamboo leaves have bilateral symmetry with parallel venation and appear similar across species. Here, we investigate whether the shape of bamboo leaves can be accurately described by a simplified Gielis equation, which consists of only two parameters (leaf length and shape) and produces a perfect bilateral shape. To test the applicability of this equation and the occurrence of bilateral symmetry, we first measured the leaf length of 42 bamboo species, examining >500 leaves per species. We then scanned 30 leaves per species that had approximately the same length as the median leaf length for that species. The leaf‐shape data from scanned profiles were fitted to the simplified Gielis equation. Results confirmed that the equation fits the leaf‐shape data extremely well, with the coefficients of determination being 0.995 on average. We further demonstrated the bilateral symmetry of bamboo leaves, with a clearly defined leaf‐shape parameter of all 42 bamboo species investigated ranging from 0.02 to 0.1. This results in a simple and reliable tool for precise determination of bamboo species, with applications in forestry, ecology, and taxonomy.

## 1 Introduction

In botany, forestry, and agriculture, understanding the development and morphogenesis of plants is of primary importance. To this end, many studies have focused on exploring potential rules of plant growth, reconstructing plant morphology, and capturing their morphogenetic dynamics. Fundamental features of plants are self‐similarity and symmetry, and all plants are in some sense repetitions of basic phytomers (Clark & Fisher, 1987). Most plants, in particular monocots, have a simple structure, a repetition of phytomers consisting of an internode and a nodal zone. At each nodal zone, a bud and protective leaf structures occur. This basic structure led to a great diversity of growth forms and to a variety of models from plant diversity (Bell & Bryan, 2008; Dabadie, Reffye, & Dinouard, 1991; Doust, 2007; Halle, 1986).

Various studies have focused on integrating the development of plants and plant organs and on simulating the dynamics of crop morphological development based on L‐systems (Fournier & Andrieu, 1999; Lindenmayer, 1968a, 1968b; Prusinkiewicz, 1999). For several important crops such as maize (Fournier & Andrieu, 1999), barley (Buck‐Sorlin & Bachmann, 2000), sorghum (Kaitaniemi, Hanan, & Room, 2000), and rice (Ding, Zhang, Zhang, Zhu, & Chen, 2011), architectural models have been linked to physiology and to measured development and growth (Chen, Jiang, Zhu, Cao, & Chen, 2004; Liu, Tang, & Qi, 2002; Watanabe et al., 2005; Zhan, Wang, Reffye, & Hu, 2001). Alternatively, specific models have been developed to address plant growth under optimal conditions that seek to reproduce the complex structure of a crop's shape. Plant growth and development is the result of the timing and spatial organization of the development of organs from a meristematic zone. In physiology, various studies have focused on timing and spatial aspects of gene expression, in relation to the formation of organs. One example is the development of lateral outgrowths of organs from apical meristems and the direct relation of this to the spatial distribution of auxin via auxin transporters (Reinhardt, Mandel, & Kuhlemeier, 2000). However, gene action or gene regulatory networks do not give a complete picture of this process. The development of such organs is dictated by mathematical and physical laws, making it no surprise that Fibonacci and Lucas series are found so frequently in phyllotaxy, both in the positioning of leaves along the stem and in the arrangement of parts in single and composite flowers (Jean, 2009). Phyllotactic patterns have also been linked to physical phenomena optimizing space utilization (Douady & Couder, 1992).

Such locally organized structures develop into organs, new phytomers or leaves, either vegetative leaves or modified leaves into floral structures. Vegetative leaves are the most important plant organs for photosynthesis, and their size and shape have an important influence on the morphogenesis and development of plants. As many plant organs vary over the growing season, it is important to identify a simple but adequate mathematical model for capturing morphological change in plants. This is not a simple task. Besides the classic phenotypic differences related to ecological habitat, there are the phenomena of heteroblasty and heterochrony to include in any accurate model. Heteroblasty is the phenomenon whereby several types of leaves occur, depending on position or age (Bell & Bryan, 2008). Heterochrony, on the other hand, describes leaf‐shape variation over time. Differences in timing can lead, for example, to simple versus compound leaves, and heterochrony has been identified as the basis for natural variation in *Cardamine hirsuta*, a close relative of *Arabidopsis*, in which differences in leaf shape were correlated to flowering time (Cartolano et al., 2015). A final difficulty to mathematical modeling of leaf shape is that it can be hard to define precise landmarks in leaves for defining morphometry. Leaves and leaflets in compound leaves in general have relatively simple shapes, but leaves can be highly variable as in *Begonia* (McLellan, 1993), *Papaya* and *Vasconcellea* (Scheldeman et al., 2011).

Vegetative leaves of bamboo, on the other hand, are relatively simple, and their structure is stable throughout the whole subfamily. The bamboos (Poaceae: Bambusoideae) consist of approximately 1,300 species of temperate, tropical woody bamboos and a tribe of herbaceous bamboos (Kelchner, 2013; Liese & Köhl, 2015; Wysocki, Clark, Attigala, Ruiz‐Sanchez, & Duvall, 2015). Unlike other grasses, bamboos are the only major lineage within the family that have adapted to and diversified within the forest habitat (Judziewicz & Clark, 2007; Judziewicz, Clark, Londono, & Stern, 1999; Liese & Köhl, 2015). The dry biomass of bamboo leaves contributes 5%–10% of the whole plant biomass for most bamboo species. Bamboo leaves consist of two major parts, a sheath and a blade, with a characteristic ligule with oral setae and auricles as appendages (Stapleton, 2012). Cauline leaves have a protective function in the development of young shoots and culms. The sheath in these types of leaves is very stiff, and the blade is greatly reduced. In contrast, foliage leaves have fully developed leaf blades and the blades are connected to the sheath via a pseudo‐petiole. This is a structure unique to all bamboo species and some large leaved grasses. These foliage leaf blades are usually linear, lanceolate or oblong‐lanceolate, with the tip long and acuminate, often scabrous and the side glabrous or softly hairy. The leaf blade is generally thinner than the culm sheath blade and often shows more marked dorsiventrality. The morphology and structure of leaves vary among species and can be used for species identification (Stapleton, 2012).

A leaf‐shape description based on Lamé curves has been used to model foliage leaves of the bamboo genus *Indocalamus* (Shi, Xu, et al., 2015). To test the applicability of this work to other bamboos, with different size and shape of leaves, in this study, we extend this modeling to a wide variety of genera and species. We propose a simple and adequate mathematical model for describing the leaf shape of bamboos, which allows for testing the bilateral symmetry of leaf shape in more than 40 different bamboo species we can have access to in the Nanjing Forestry University campus. In addition, we will show that this model also can be used to assist in identifying bamboo species.

## 2 Materials and Methods

### 2.1 Experimental design

To test the generality of bilateral symmetry in bamboo leaves, we collected 42 bamboo species from the Nanjing Forestry University campus (32°04′34.53′′N, 118°48′42.06′′E; Table S1). In the Nanjing Forestry University campus, the environmental factors can be regarded as constants because of low spatial heterogeneity. Nanjing belongs to the subtropical monsoon climate, with mean annual precipitation of 1,058 mm (±237.5 mm) and a mean annual temperature of 15.6°C (±0.7°C). Mean minimum annual temperature was −8.6°C, and the mean maximum annual temperature was 37.4°C (based on the climate data of 1951–2012 downloaded from China Climate Data Online, www.data.cma.cn).

Table S1 lists the official names of the sampled species according to the Flora of China (Wu, Raven, & Hong, 2006). If no entry was found in the Flora of China, the name was selected from Bamboos of the World (Ohrnberger, 1999). For every species, we randomly chose more than 500 leaves from different individuals and measured their lengths to check the normality of leaf length distribution and to calculate the median of leaf lengths. Then, 30 leaves with length ≈ the median were collected, scanned, and analyzed.

### 2.2 The Gielis equation

**Figure 1:**
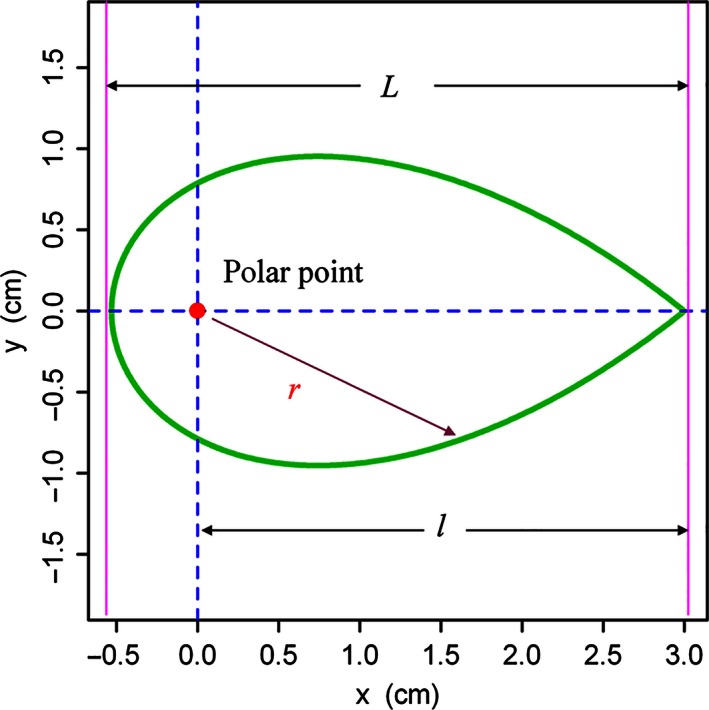
Leaf shape produced by the simplified Gielis equation

Let us assume (*x*,* y*) be the Cartesian coordinates of a point on the boundary of a leaf. We can transform this point into the polar coordinates as the following:

(1) $$\left\{\begin{array}{c} x=r\cdot \cos\varphi \\ y=r\cdot \sin\varphi \end{array}\right.,$$

where *r* is the polar radius (i.e., the distance between the polar origin and the point; Fig. 1), and φ the angle of the radial vector.

For testing the bilateral symmetry of plant leaves, we put forward a simplified version of the Gielis equation (Gielis, 2003a, 2003b; Gielis & Gerats, 2004).

(2) $$r=\frac{l}{{\left(|\cos\frac{\varphi }{4}|+|\sin\frac{\varphi }{4}|\right)}^{1/n}}.$$

Comparing this with the general Gielis equation (Gielis, 2003a)

(3) $$r=\frac{1}{{\left\{{\left(|\frac{1}{a}\cos(\varphi \frac{m}{4})|\right)}^{n_{2}}+{\left(|\frac{1}{b}\sin(\varphi \frac{m}{4})|\right)}^{n_{3}}\right\}}^{1/n_{1}}},$$

where *m*,* a*,* b*,* n*_1_, *n*_2,_ and *n*_3_ are constants, we find symmetry parameter *m *=* *1 and exponents on cosine and sine terms both equal to 1 (in the general Gielis equation, the parameter *m* and the exponents can be other values; see Gielis (2003a, 2003b) for details). The parameter *l* is a constant value, namely the distance from the polar origin to the leaf tip. We have *r = l* when φ = 0. The number *n* is a parameter that can determine the overall ratio of the leaf width to length and is independent from the absolute leaf length; hereafter, we refer to *n* as the leaf‐shape parameter. The leaf length (*L*) is the sum of *r* when φ = 0 and when φ = π (see Fig. 1):

(4) $$L=\left(1+2^{-\frac{1}{2n}}\right)\cdot l.$$

It is apparent that this simplified Gielis equation (i.e., eq. (2)) could produce the bilateral symmetry as r(φ)=r(−φ).

In practice, there are two issues for estimating the two parameters in the above simplified Gielis equation when using the leaf‐shape data extracted from a scanned image of a real leaf: (1) the polar origin usually deviates from the coordinate point of (0, 0); and (2) the angle between the line passing from the polar origin to the leaf tip and the actual horizontal axis usually also deviates from 0°. To solve these two issues, we took the method of Shi, Huang, et al. (2015) and Shi, Xu, et al. (2015) by estimating three parameters: *x*_0_, *y*_0_, and θ. The first two parameters reflect the shift of coordinates from (0, 0) to (*x*_0_, *y*_0_), and the last parameter reflects the angle change in the horizontal axis from 0° to θ. We used the optimization algorithm proposed by Nelder and Mead (1965) to fit the parameters of the simplified Gielis equation using the “optim” function in R software (R Core Team 2015).

### 2.3 Data analysis

For every species, we randomly chose more than 500 matured foliage leaves and measured their lengths, as the distance from the leaf bottom to the tip. We need to choose the leaves whose lengths can reflect the generality of a population. If leaf length follows a normal distribution (namely a symmetric density distribution curve), the mean or median both can represent the generality of a population; if its density distribution curve is skewed (that has a long left or right tail) like the Weibull distribution, the median is better than the mean in reflecting the generality of a population. The Shapiro–Wilk test (Quinn & Keough, 2002) was used to check the normality of leaf lengths, and the Kolmogorov–Smirnov test (Quinn & Keough, 2002) for examining whether the leaf length follows the Weibull distribution. We then chose 30 leaves per species of approximately equal to the median of leaf lengths and scanned them to extract Cartesian coordinates on the leaf boundary (Shi, Huang, et al., 2015). The bilateral symmetry of bamboo leaves was tested by the goodness of fit of the simplified Gielis equation. We also tested whether the bamboo species that are more closely related in taxon have smaller difference in the leaf‐shape parameter. Tukey's HSD (honestly significant difference) test (Quinn & Keough, 2002) was used to examine the pairwise difference of leaf‐shape parameters between different bamboo species.

## 3 Results

### 3.1 Leaf length

**Figure 2:**
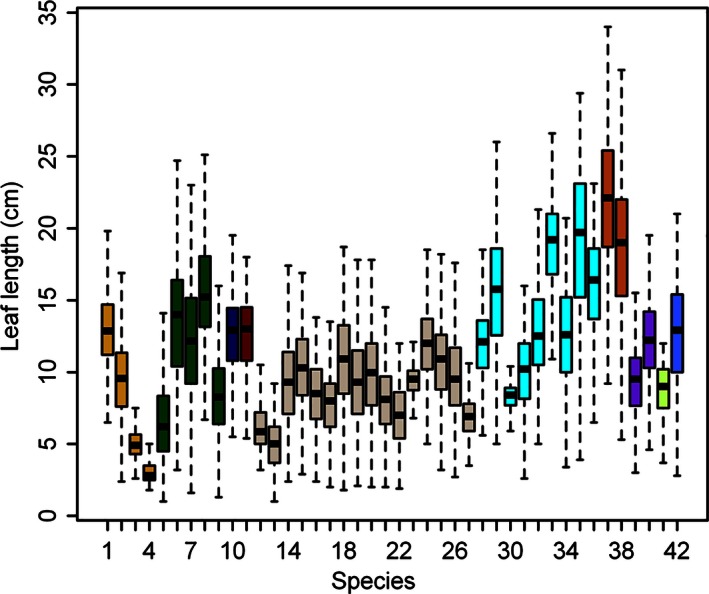
Comparison of leaf lengths for 42 bamboo species

The length of the leaves of these 42 bamboo species follows the Weibull distribution rather than the normal distribution (Table S2, Fig. 2). We found leaf length to differ significantly among genera and among species within the same genus. Sampled leaves could be very small, <5 cm length in most species, while the maximum length ranged up to 35 cm for *Pseudosasa amabilis* var. *convexa*, but for most species was below 25 cm. While the variation in leaf length of the genus *Phyllostachys* was less than the variation of the genus *Pleioblastus*,* Phyllostachys* is a narrowly defined genus with restricted natural geographic distribution while that of *Pleioblastus* is very broad, encompassing bamboos from different temperate zones of the world.

### 3.2 Goodness of fit and bilateral symmetry

**Figure 3:**
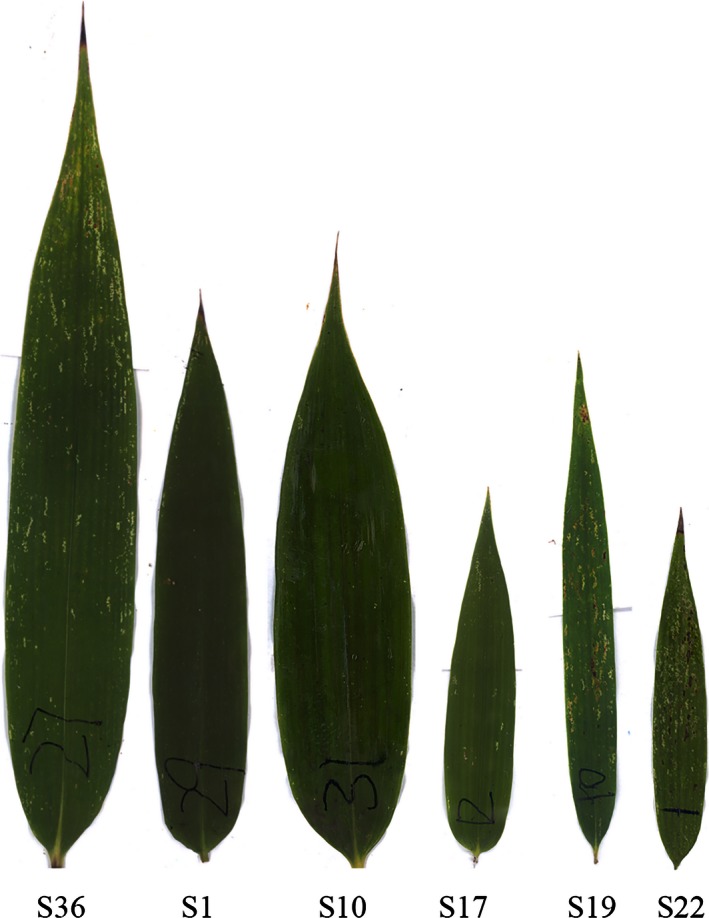
Scanned leaf images of six bamboo species. S36: *Pleioblastus yixingensis*; S1: *Bambusa emeiensis* var. *viridiflavus*; S10: *Indosasa shibataeoides*; S17: *Phyllostachys bissetii*: S19: *Phyllostachys edulis*; and S22: *Phyllostachys edulis “Gracilis”*

**Figure 4:**
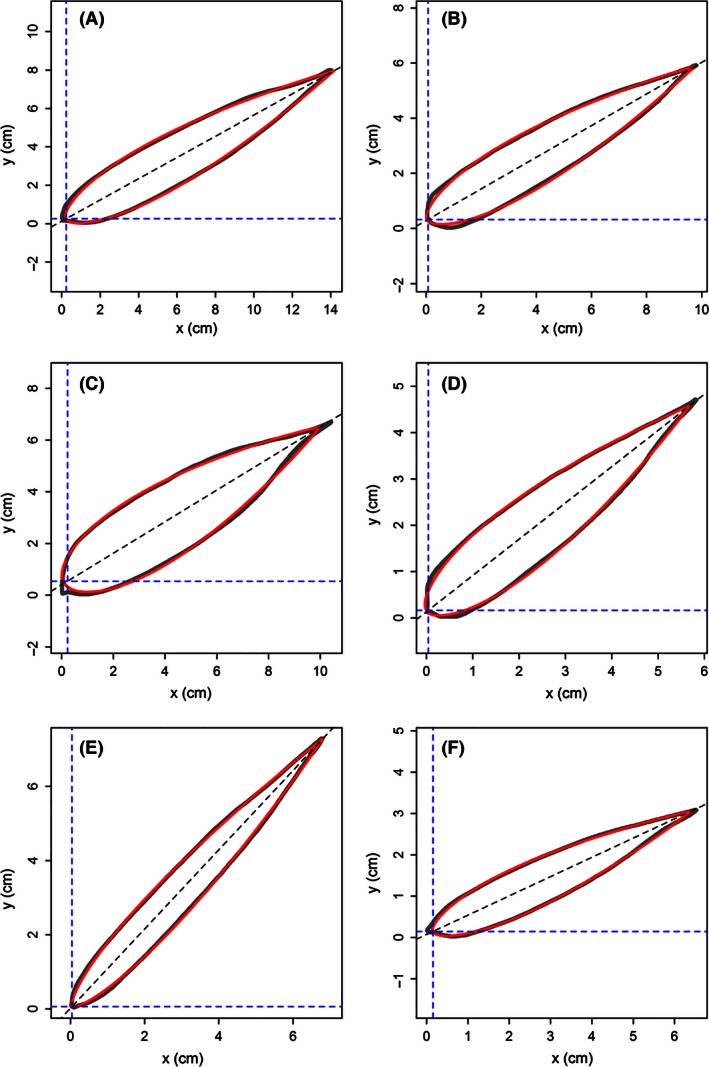
Comparison between scanned leaf profile (gray line) and predicted leaf profile (red line) from the simplified Gielis equation. (A) *Pleioblastus yixingensis*; (B) *Bambusa emeiensis* var. *viridiflavus;* (C) *Indosasa shibataeoides*; (D) *Phyllostachys bissetii*; (E) *Phyllostachys edulis*; (F) *Phyllostachys edulis* “Gracilis”

**Figure 5:**
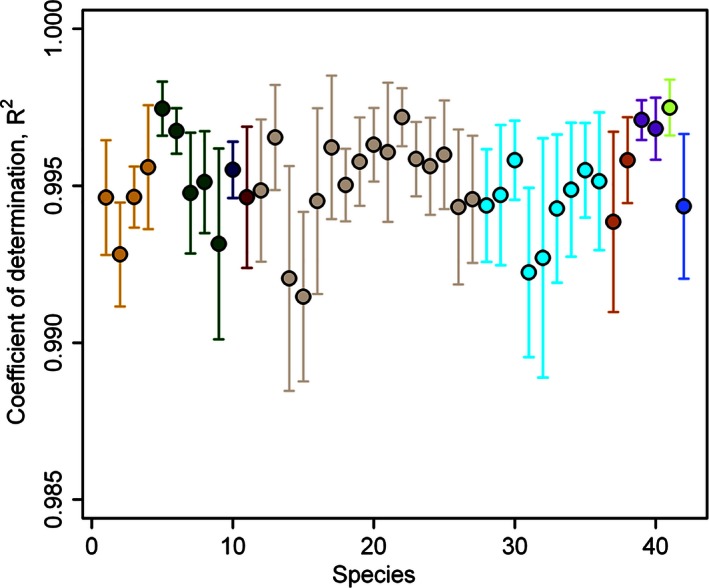
Coefficients of determination for fitting the leaf shapes of 42 bamboo species to the simplified Gielis equation

**Figure 6:**
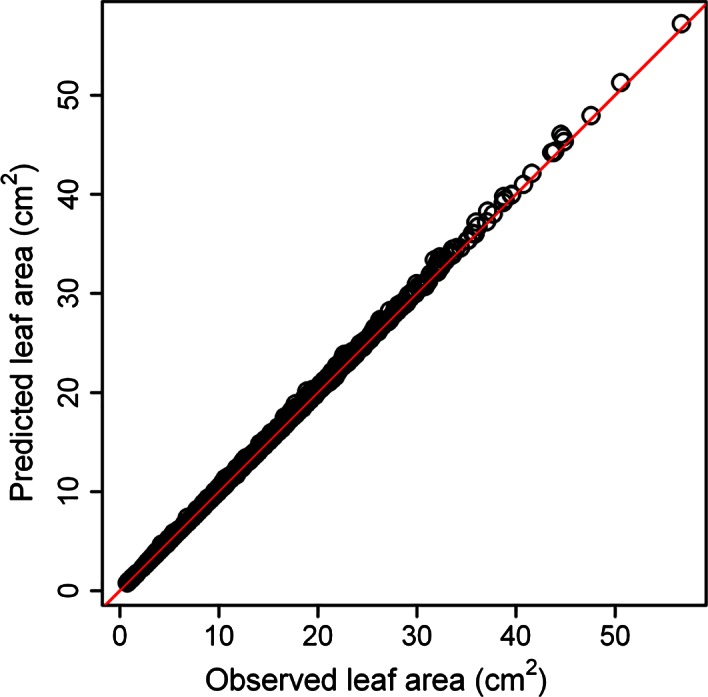
Comparison between the real and predicted leaf areas estimated from the simplified Gielis equation. The red straight line represents *y = x*, the small open circles are made up of the actual leaf areas and the predicted leaf areas

The leaf shape of all bamboo species (see Fig. 3 for examples) was described by the simplified Gielis equation well (see Table S3), with the results for six bamboo species illustrated in Fig. 4. The predicted leaf shape matched the observed leaf shape well for these six bamboo species. The goodness of fit also provides convincing evidence of bilateral symmetry in bamboo leaves, with all coefficients of determination higher than 0.980 (Fig. 5). The real leaf area fits extremely well with the predicted leaf area (Fig. 6), with the regression straight line deviated only trivially from the straight line of *y = x*.

**Figure 7:**
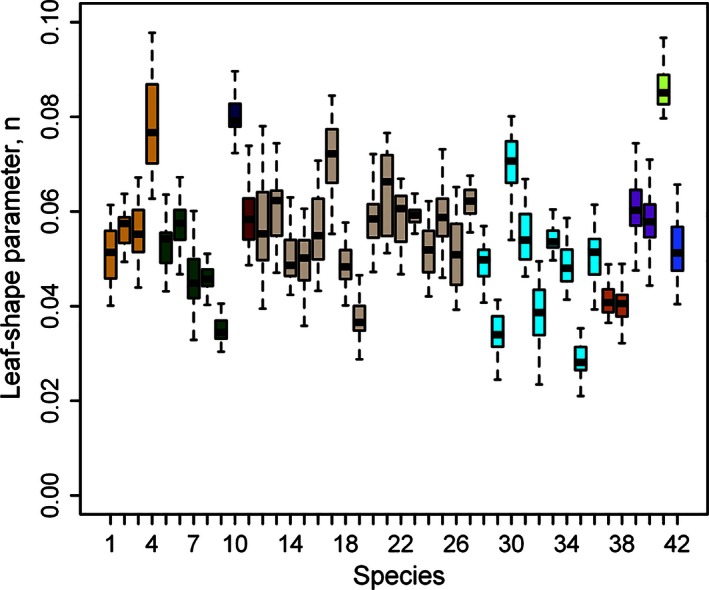
Comparison of the estimated leaf‐shape parameters for 42 bamboo species

The variation of leaf‐shape parameters for these 42 species is illustrated in Fig. 7. Although there were significant differences in the leaf‐shape parameters among species from different genera or from the same genus (Table S4), the calculated leaf‐shape parameters for these 42 species range only from 0.02 to 0.1. The lower values are for shapes of a more linear‐lanceolate type, such as leaves of *Pleioblastus chino*,* Pleioblastus simonii* f. *heterophyllus, Pleioblastus gramineus* f. *monstrispiralis*,* Chimonobambusa tumidissinoda,* and *Phyllostachys edulis*. Leaves described by the higher values of the shape parameter *n* were slightly broader in shape, such as those of *Shibataea chinensis, Indosasa shibaeatoides* (Fig. 3), and *Bambusa multiplex* var. *riviereorum*. The remaining leaves fell broadly within the range of shape parameters 0.03–0.08.

## 4 Discussion

### 4.1 Capturing the diversity of bamboo leaves

Our results corroborate findings for some dwarf bamboos (Shi, Xu, et al., 2015) and suggest that modeling with the modified Gielis equation that has a reduced number of parameters is clearly applicable to a wide variety of temperate bamboo genera and species with lanceolate leaves. A range of shapes and sizes from the small leaves of *Bambusa multiplex var. riviereorum* to the large leaves of *Indocalamus victorialis* and from the linear‐lanceolate leaves of *P. linear* to the broader leaves of *Shibataea* can all be efficiently modeled with one equation with only two parameters. The 42 bamboo species examined in this study were randomly chosen from the bamboo garden of Nanjing Forestry University. Although all are Asian bamboos, both temperate and tropical, the mix was chosen to be representative of the whole subfamily. While the only tropical woody bamboos in this study are the ornamental *Bambusa* species, our findings can certainly be extended to most, if not all, other tropical bamboos.

Leaf length of these bamboo species shows significant differences between species, but in general, the shape parameter varies little across species, from 0.02 to 0.1. Also, three *Indocalamus* species had shape parameter values between 0.04 and 0.07 (Shi, Xu, et al., 2015). The only exception is *Indosasa victorialis*, where the value is above 0.1. Although the leaf sizes (length and width) of the 42 bamboo species examined in this study and the four species studied in the paper (Shi, Xu, et al., 2015) differ considerably, their leaf shapes vary little and show general lanceolate characteristics. Our results showed that the simplified Gielis equation delineates leaf shapes of different bamboo species extremely well. Whereas in the past bamboo leaf blades were characterized by length and width with additional qualitative characteristic such as linear‐lanceolate or oblong‐lanceolate, we now have a clear quantitative number for a qualitative characteristic. Molecular analysis and genetic assignments usually are used to study differentiation and hybridization of some closely related species and secondary for identification of established species.

Our results also demonstrated the bilateral symmetry of the leaf shape of all these bamboos. The occurrence of bilateral symmetry might be helpful for the transportation of nutrients and water from branches to leaves. The greater the goodness of fit is when using the Gielis equation, the stronger the bilateral symmetry of the leaves, thereby confirming our original assumption that r(φ)=r(−φ). The interpretation of this symmetry is global, concerning the whole leaf, rather than local. Carefully examining the base of the leaf and the vascular bundles emanating from the pseudo‐petiole (Fig. 3), the bases are rarely perfectly symmetric, but on average for the population the leaves are bilaterally symmetric (Fig. 3), and this symmetry is a guiding basis of development. The fact that bilateral symmetry and leaf blades connected to the sheath part by a pseudo‐petiole are typical of all bamboos foliage leaf blades potentially reflects the evolutionary stability of this solution. Indeed, bamboos are one of very few groups in the grass family that have evolved in forests, not in open areas, and can reach quite large sizes (up to 20 m for temperate bamboos and up to 30 m for tropical bamboos). Hollow and flexible culms and branches, protected during development with very stiff culms or branch sheaths on the one hand, and leaf blades that are connected through pseudo‐petioles allowing for torsion on the other hand contribute to the evolutionary success of woody and herbaceous bamboos. The anatomy of bamboo leaves is very stable throughout the subfamily (Brandis, 1907). It is remarkable that the pseudo‐petiole, a specialized structure in all bamboos (and only some grasses with larger leaves), has hardly been studied. The course of the vascular bundle and the possible presence of pulvini should be investigated, as pseudo‐petioles allow bamboos to adjust the orientation of the blades toward the sun or to ease torsion caused by wind or snow loads.

The model established in this article can be extended to many more bilaterally symmetric foliage leaves. Bamboo leaves (and grass leaves in particular) show parallel venation characteristics of monocots. The venation of bamboo leaves is parallel with three orders, namely the midrib, secondary veins, and tertiary veins. In bamboos, adjacent veins are connected by transverse distinct veinlets, visible in temperate bamboos but hidden in tropical species (Brandis, 1907). In particular, bamboo leaves show multicostate parallel convergent venation whereby the main vascular systems diverge from the pseudo‐petiole and converge at the apex of the leaves. This leads to a clear bilateral symmetry, and the model can also be used for many other monocots with similar venation. However, the venation of a unicostate type in monocot leaves, with one main vein and branching along the vein as in banana leaves, is also clearly symmetric and can be modeled in the same way. The model can be extended to all bilaterally symmetric leaves, both in simple and compound leaves, and including petals in composite or simple flowers.

Compared to the general equation with six parameters, the symmetry parameter *m *=* *1, the exponents *n*_2_ and *n*_3_ are both equal to one, and *n*_1_ = *n,* the number of is reduced here to 1, namely the exponent *n*. With the general Gielis equation and more parameters, other leaf shapes such as orbicular, hastate, elliptical, cordate, and others (Gielis, 2003a; Wang, 2007) can also be described efficiently, either directly as transformation of the circle or indirectly as modifications of cardioid or similar functions (Gielis, 2003a; Wang, 2007). For plants with nonsymmetric leaves, more complicated mathematical models are needed, such as elliptic Fourier analysis or summations of eq. (2) into *k*‐type functions (Gielis et al., 2012). For asymmetrical leaf bases, adjusting the condition r(φ)=r(−φ) could be considered. The original Gielis equation could then be a first choice for describing nonsymmetric leaf shapes with smooth margins. The shape of petioles, typically concave for large leaves, has also been modeled efficiently with the same approach (Faisal, Abad, Hristozov, & Pasini, 2010). We therefore recommend simplifying the original Gielis equation contextually for different purposes.

### 4.2 Leaf development models and the future trends

Leaf‐shape models for species of the grass family have been proposed to address development (Dornbusch, Watt, Baccar, Fournier, & Andrieu, 2009; Dornbusch, Watt, Baccar, Fournier, & Andrieu, 2011; Zhu et al., 2009). In particular, Dornbusch et al. (2009) analyzed the leaf shapes of wheat, barley, and maize using an empirical shape model proposed in wheat (Dornbusch et al., 2011) and based on three‐dimensional parameters and three shape parameters. They found that varying conditions during growth will affect leaf dimensions but not leaf shapes. The quantification of leaf expansion in *Miscanthus* species and *Brachypodium*, in particular duration and timing of leaf growth in relation to environmental parameters, allows for distinguishing between closely related genotypes (Shi, Chen, Hui, & Grissino‐Mayer, 2016; Voorend et al., 2014). Along with leaf expansion, the model proposed by Dornbusch et al. (2011) could also be used to quantify cell elongation in leaves.

There are very few geometrical models for modeling leaves. Many models of planar plant leaves use ellipses as a starting point. A multiparametric model was developed for the description of the axial‐symmetric convex pentagon of grass leaf shape (Dornbusch et al., 2011). In contrast, for modeling bamboo leaves with the Gielis equation, we now need only two model parameters, with extremely high goodness of fit for all species (>0.98), and confirming the bilateral symmetry of bamboo leaves. In this study, quantitative measurements were performed for 500 leaves, and scans and comparison with models for 30 leaves per species. As the scanned leaves per species were of similar size, this range should also reflect differences in leaf age and positioning, confirming the Weibull distribution also observed by Shi, Xu, et al. (2015). This distribution originates from the study of different particle sizes in crushed particles, sand, or volcanic ash, following power laws. This distribution implies that leaf length in bamboo follows a power law.

One advantage of our model is that the associated characteristics (perimeter, area, polar moment of inertia) can be computed directly from the analytical expressions of these characteristics for leaf‐shape variation, by observing cross sections along the petiole (Faisal et al., 2010) or stems (Dornbusch et al., 2009).

While little quantitative information is available on the development of bamboo leaf blades, the development of these blades is qualitatively quite simple. After elongation of the culm or branching, the leaves develop in an acropetal way (Banik, 2015). They develop along the axis of the midrib fully parallel to the main axis of the branch, inside the preceding sheath, and emerge through the upper opening of that preceding sheath. When they emerge they unroll until they are planar. This process of convolute vernation (Brandis, 1907) involves the turgor state of the bulliform cells, the same process that occurs when leaf blades roll up in dry conditions. Certainly, this precise process requires further research over the whole bamboo subfamily.

## 5 Conclusions

The original Gielis formula has six parameters, as a transformation on any plane curve. This increases the potential for modeling leaves or other botanical shapes such as tree rings (Lei & Koike, 1998; Shi, Huang, et al. 2015; Shi, Xu, et al. 2015) and petioles (Faisal et al. 2010). It may become a uniform approach for studying leaves in plants or other shapes and could be better than existing models, as shown by using the Akaike information criterion (Shi, Huang, et al., 2015). From a geometric and mathematical point of view, it is a generalization of the Pythagorean theorem, retaining its structure with separated variables, useful for studying development from an applied mathematics point of view (Caratelli et al., 2009; Gielis et al., 2012).

Here, a simplified version of the Gielis equation was shown to be an excellent model for describing the foliage leaf blades of bamboo with lanceolate characteristics and bilateral symmetry, corroborating earlier findings on four species of *Indocalamus* (Shi, Xu, et al., 2015). The present study demonstrated this bilateral symmetry in leaves of 42 bamboo species, including four sympodial (clustered) species, 16 monopodial (scattered) species, and 22 mixed species (that have the characteristics of both clustering and scattering). In this study, this was performed by measuring thousands of leaves to determine lengths and a subset of over one thousand to determine shape parameters.

Only two parameters are involved, one for length and the other for shape, accounting for full variations in shape and dimensions (compared to three‐dimensional and three shape parameters in wheat, barley, and maize (Dornbusch et al., 2011)). Keeping the shape parameter constant and modify the length parameter according to the species' minimum and maximum leaf lengths and the specific Weibull distributions should suffice to faithfully regenerate leaf shapes and images encompassing the variation on a plant within a given species. As the leaf shape of plants can be affected by many factors such as genetics, cultivar, and growing conditions, future research could focus on explaining the variation of leaf shape in bamboos at different ages and under different controlled environments such as sites with different levels of nutrients. Our method will allow for the building of reliable databases of genotypes for bamboo studies worldwide, supplementing molecular markers (Gielis, Everaert, Goetghebeur, & Deloose, 1995; Hodkinson, Renvoize, Chonghaile, Stapleton, & Chase, 2000; Lin, Ruan, Lou, Guo, & Fang, 2009; Schiessl, Kausika, Southam, Bush, & Sablowski, 2012; Suyama, Obayashi, & Hayashi, 2000) for precise identification.

## Funding Information

This work was financially supported by the Key Project of National Science & Technology Ministry (2016YFD0600901), the National Postdoctoral Fund of China (2014M560427), the National Natural Science Foundation for Young Scholars of China (31400348 and 31000294), and the Priority Academic Program Development of Jiangsu Higher Education Institutions. Cang Hui is supported by the National Research Foundation of South Africa (81825 and 76912) and the Australian Research Council (Discovery Project DP150103017).

## Conflict of Interest

None declared.
