# Supplementary material for: A geometrical model for testing bilateral symmetry of bamboo leaf with a simplified Gielis equation
Source: Ecol Evol. 2016 Sep 1;6(19):6798–806. doi: 10.1002/ece3.2407 (PMC5513222; doi:10.1002/ece3.2407)
Supplement: Supplementary file 1 [file ECE3-6-6798-s001.docx]

**Supplementary materials.** Supplementary tables

**Table S1.** List of the 42 bamboo species

| Species code | Latin name |
| --- | --- |
| 1 | *Bambusa emeiensis* var. *viridiflavus* Hsuen et Yi |
| 2 | *Bambusa multiplex* (Loureiro) Raeuschel ex Schultes & J. H. Schultes |
| 3 | *Bambusa multiplex* f. *fernleaf* (R. A. Young) T. P. Yi, |
| 4 | *Bambusa multiplex* var. *riviereorum* Maire |
| 5 | *Chimonobambusa marmorea* f. *variegata* (Mitford) Makino |
| 6 | *Chimonobambusa neopurpure*a Yi |
| 7 | *Chimonobambusa quadrangularis* (Franceschi) Makino |
| 8 | *Chimonobambusa sichuanensis* (T. P. Yi) T. H. Wen |
| 9 | *Chimonobambusa tumidissinoda* Hsueh & YI, ex. Ohrnb*.* |
| 10 | *Indosasa shibataeoides* McClure |
| 11 | *Oligostachyum sulcatum* Z. P. Wang & G. H. Ye |
| 12 | *Phyllostachys arcana* cv. luteosulcata McClure*,* |
| 13 | *Phyllostachys aurea* Carrière ex Rivière & C. Rivière |
| 14 | *Phyllostachys aureosulcata* McClure |
| 15 | *Phyllostachys aureosulcata* f. pekinensis J.L. Lu |
| 16 | *Phyllostachys aureosulcata* f. spectabilis C.D. Chu & C.S. Chao |
| 17 | *Phyllostachys bissetii* McClure |
| 18 | *Phyllostachys dulcis* McClure |
| 19 | *Phyllostachys edulis* (Carrière) J. Houzeau |
| 20 | *Phyllostachys glauca* McClure |
| 21 | *Phyllostachys heteroclada* Oliver |
| 22 | *Phyllostachys edulis 'Gracilis' (W.Y. Hsiung C.S. Chao) & S. Renvoize* |
| 23 | *Phyllostachys nidularia* Munro |
| 24 | *Phyllostachys nigra* f. *henonis (Mitford) Muroi* |
| 25 | *Phyllostachys nigra* (Loddiges ex Lindley) Munro |
| 26 | *Phyllostachys sulphurea* var. *viridis* (Carrière) Rivière & C. Rivière |
| 27 | *Phyllostachys violascens* (Carrière) Rivière & C. Rivière |
| 28 | *Pleioblastus argenteostriatus* (Regel) Nakai |
| 29 | *Pleioblastus chino* (Franchet & Savatier) Makino |
| 30 | *Pleioblastus distichus* (Mitford) Nakai |
| 31 | *Pleioblastus fortunei* (Van Houtte) Nakai |
| 32 | *Pleioblastus gramineus* f. *monstrispiralis* (Y. Okada) Muroi & H.Hamada |
| 33 | *Pleioblastus kongosanensis* f. *aureostriatus Muroi & Y. Tanake* |
| 34 | *Pleioblastus maculatus* (McClure) C. D. Chu & C. S. Chao |
| 35 | *Pleioblastus simonii* f. *heterophyllus* (Makino & Shirasawa) Muroi |
| 36 | *Pleioblastus yixingensis* S. L. Chen & S. Y. Chen |
| 37 | *Pseudosasa amabilis* var. *convexa* Z. P. Wang & G. H. Ye |
| 38 | *Pseudosasa japonica* var. tzutsumiana (Siebold & Zuccarini ex Steudel) Makino ex Nakai |
| 39 | *Semiarundinaria densiflora* (Rendle) T. H. Wen |
| 40 | *Semiarundinaria sinica* T. H. Wen |
| 41 | *Shibataea chinensis* Nakai |
| 42 | *Sinobambusa tootsik* (Makino) Makino |

**Table S2.** Leaf lengths of the 42 bamboo species

| Species code | Median | Mean | Standard error | Sample size | *p*-value  (Normal distribution) | *p*-value  (Weibull distribution) |
| --- | --- | --- | --- | --- | --- | --- |
| 1 | 12.85 | 13.20 | 3.09 | 500 | < 0.01 | < 0.01 |
| 2 | 9.55 | 9.70 | 2.89 | 500 | < 0.01 | 0.046 |
| 3 | 4.90 | 4.98 | 1.02 | 500 | 0.077 | 0.017 |
| 4 | 2.80 | 3.07 | 0.89 | 500 | < 0.01 | < 0.01 |
| 5 | 6.20 | 6.51 | 2.64 | 500 | < 0.01 | 0.137 |
| 6 | 14.00 | 13.80 | 4.25 | 500 | 0.123 | 0.529 |
| 7 | 12.15 | 12.10 | 4.29 | 500 | < 0.01 | 0.188 |
| 8 | 15.20 | 15.41 | 3.64 | 500 | 0.016 | 0.095 |
| 9 | 8.25 | 8.34 | 2.81 | 500 | < 0.01 | 0.385 |
| 10 | 12.90 | 12.62 | 2.74 | 500 | < 0.01 | 0.413 |
| 11 | 13.00 | 12.51 | 2.80 | 500 | < 0.01 | 0.169 |
| 12 | 5.85 | 6.17 | 1.54 | 500 | < 0.01 | < 0.01 |
| 13 | 5.00 | 4.98 | 1.60 | 500 | < 0.01 | 0.266 |
| 14 | 9.30 | 9.27 | 2.97 | 700 | 0.013 | 0.661 |
| 15 | 10.30 | 10.21 | 2.95 | 500 | < 0.01 | 0.469 |
| 16 | 8.50 | 8.43 | 2.32 | 500 | < 0.01 | 0.580 |
| 17 | 8.00 | 7.64 | 2.38 | 1051 | < 0.01 | < 0.01 |
| 18 | 10.90 | 10.69 | 3.43 | 500 | < 0.01 | 0.210 |
| 19 | 9.30 | 9.27 | 2.88 | 500 | 0.017 | 0.184 |
| 20 | 9.95 | 9.93 | 3.28 | 500 | 0.651 | 0.876 |
| 21 | 8.10 | 8.15 | 2.90 | 700 | < 0.01 | < 0.01 |
| 22 | 7.00 | 6.98 | 2.15 | 500 | < 0.01 | 0.643 |
| 23 | 9.50 | 9.41 | 1.11 | 500 | < 0.01 | 0.093 |
| 24 | 12.00 | 11.77 | 2.88 | 500 | < 0.01 | 0.497 |
| 25 | 10.90 | 10.74 | 2.69 | 500 | 0.276 | 0.814 |
| 26 | 9.50 | 9.88 | 3.41 | 500 | < 0.01 | < 0.01 |
| 27 | 6.90 | 6.90 | 1.47 | 602 | < 0.01 | 0.032 |
| 28 | 12.10 | 11.96 | 2.62 | 500 | 0.163 | 0.279 |
| 29 | 15.75 | 15.59 | 4.20 | 500 | 0.069 | 0.707 |
| 30 | 8.40 | 8.29 | 0.94 | 499 | < 0.01 | 0.021 |
| 31 | 10.20 | 9.92 | 2.56 | 500 | < 0.01 | 0.158 |
| 32 | 12.50 | 12.89 | 3.28 | 500 | < 0.01 | 0.062 |
| 33 | 19.20 | 18.61 | 3.26 | 500 | < 0.01 | 0.079 |
| 34 | 12.60 | 12.51 | 3.56 | 500 | < 0.01 | 0.600 |
| 35 | 19.70 | 18.56 | 5.75 | 616 | < 0.01 | < 0.01 |
| 36 | 16.40 | 15.89 | 3.70 | 872 | < 0.01 | 0.012 |
| 37 | 22.10 | 21.77 | 4.95 | 805 | < 0.01 | 0.648 |
| 38 | 19.00 | 18.39 | 5.04 | 890 | < 0.01 | 0.020 |
| 39 | 9.50 | 9.30 | 2.34 | 500 | < 0.01 | 0.054 |
| 40 | 12.20 | 12.08 | 2.91 | 500 | < 0.01 | 0.759 |
| 41 | 9.00 | 8.70 | 1.82 | 700 | < 0.01 | < 0.01 |
| 42 | 12.90 | 12.61 | 3.93 | 500 | < 0.01 | 0.425 |

Sample size represents the number of sampled leaves,

**Table S3.** Fitted results from the simplified Gielis equation (see the xls file)

**Table S4.** Comparison of the leaf-shape parameter of 42 bamboo species (Tukey’s HSD test)

| Species code | Mean | Standard error | Sample size | Significance^*^ |
| --- | --- | --- | --- | --- |
| 1 | 0.0509 | 0.0060 | 30 | ijklmn |
| 2 | 0.0567 | 0.0037 | 30 | fghi |
| 3 | 0.0554 | 0.0058 | 31 | fghijk |
| 4 | 0.0789 | 0.0102 | 30 | b |
| 5 | 0.0523 | 0.0049 | 33 | hijklm |
| 6 | 0.0573 | 0.0052 | 30 | fgh |
| 7 | 0.0452 | 0.0060 | 30 | no |
| 8 | 0.0466 | 0.0041 | 30 | mno |
| 9 | 0.0359 | 0.0052 | 30 | pq |
| 10 | 0.0801 | 0.0037 | 32 | ab |
| 11 | 0.0590 | 0.0066 | 30 | efg |
| 12 | 0.0568 | 0.0108 | 30 | fghi |
| 13 | 0.0609 | 0.0074 | 30 | ef |
| 14 | 0.0503 | 0.0062 | 30 | jklmn |
| 15 | 0.0499 | 0.0067 | 30 | jklmn |
| 16 | 0.0560 | 0.0076 | 30 | fghij |
| 17 | 0.0718 | 0.0071 | 30 | c |
| 18 | 0.0488 | 0.0043 | 30 | lmn |
| 19 | 0.0382 | 0.0057 | 30 | pq |
| 20 | 0.0594 | 0.0078 | 30 | efg |
| 21 | 0.0643 | 0.0085 | 30 | de |
| 22 | 0.0576 | 0.0082 | 30 | fgh |
| 23 | 0.0594 | 0.0025 | 30 | efg |
| 24 | 0.0519 | 0.0057 | 30 | hijklm |
| 25 | 0.0590 | 0.0068 | 30 | efg |
| 26 | 0.0516 | 0.0078 | 30 | hijklm |
| 27 | 0.0610 | 0.0057 | 30 | ef |
| 28 | 0.0496 | 0.0046 | 30 | klmn |
| 29 | 0.0344 | 0.0041 | 30 | qr |
| 30 | 0.0692 | 0.0072 | 30 | cd |
| 31 | 0.0552 | 0.0061 | 30 | fghijk |
| 32 | 0.0383 | 0.0063 | 30 | pq |
| 33 | 0.0536 | 0.0044 | 30 | ghijkl |
| 34 | 0.0488 | 0.0047 | 30 | lmn |
| 35 | 0.0288 | 0.0037 | 30 | r |
| 36 | 0.0507 | 0.0055 | 30 | ijklmn |
| 37 | 0.0413 | 0.0034 | 30 | op |
| 38 | 0.0410 | 0.0058 | 30 | op |
| 39 | 0.0607 | 0.0066 | 30 | ef |
| 40 | 0.0576 | 0.0060 | 30 | fgh |
| 41 | 0.0858 | 0.0039 | 30 | a |
| 42 | 0.0519 | 0.0066 | 30 | hijklm |

^*^ The letters in the column of significance represent the significance of difference in the leaf-shape parameter among different bamboo species. The critical *p*-value is 0.05.
